# iPSC-derived breast cancer models: advancing the study of *BRCA1*-driven tumorigenesis

**DOI:** 10.1093/lifemedi/lnag006

**Published:** 2026-02-02

**Authors:** Pengguihang Zeng, Zhuheng Zhang, Xinyi Liu, Junjun Ding

**Affiliations:** Department of Rehabilitation Medicine, The Seventh Affiliated Hospital, Zhongshan School of Medicine, Sun Yat-sen University, Guangzhou 510080, China; School of Basic Medical Sciences, Guangdong Medical University, Guangzhou 511436, China; Frontiers Medical Center, Tianfu Jincheng Laboratory, Department of Gynecology and Obstetrics, West China Second Hospital, West China Biomedical Big Data Center, West China Hospital/West China School of Medicine, Sichuan University, Chengdu 610041, China; Shenzhen Eye Hospital, Shenzhen Eye Medical Center, Southern Medical University, Shenzhen 510086, China; Department of Rehabilitation Medicine, The Seventh Affiliated Hospital, Zhongshan School of Medicine, Sun Yat-sen University, Guangzhou 510080, China; School of Basic Medical Sciences, Guangdong Medical University, Guangzhou 511436, China; Frontiers Medical Center, Tianfu Jincheng Laboratory, Department of Gynecology and Obstetrics, West China Second Hospital, West China Biomedical Big Data Center, West China Hospital/West China School of Medicine, Sichuan University, Chengdu 610041, China; Shenzhen Eye Hospital, Shenzhen Eye Medical Center, Southern Medical University, Shenzhen 510086, China; Department of Rehabilitation Medicine, The Seventh Affiliated Hospital, Zhongshan School of Medicine, Sun Yat-sen University, Guangzhou 510080, China; School of Basic Medical Sciences, Guangdong Medical University, Guangzhou 511436, China; Frontiers Medical Center, Tianfu Jincheng Laboratory, Department of Gynecology and Obstetrics, West China Second Hospital, West China Biomedical Big Data Center, West China Hospital/West China School of Medicine, Sichuan University, Chengdu 610041, China; Shenzhen Eye Hospital, Shenzhen Eye Medical Center, Southern Medical University, Shenzhen 510086, China; Department of Rehabilitation Medicine, The Seventh Affiliated Hospital, Zhongshan School of Medicine, Sun Yat-sen University, Guangzhou 510080, China; School of Basic Medical Sciences, Guangdong Medical University, Guangzhou 511436, China; Frontiers Medical Center, Tianfu Jincheng Laboratory, Department of Gynecology and Obstetrics, West China Second Hospital, West China Biomedical Big Data Center, West China Hospital/West China School of Medicine, Sichuan University, Chengdu 610041, China; Shenzhen Eye Hospital, Shenzhen Eye Medical Center, Southern Medical University, Shenzhen 510086, China

The advent of induced pluripotent stem cells (iPSCs) has created a powerful platform for building human disease models. Since Yamanaka’s pioneering demonstrations in 2006–2007 that mouse and human fibroblasts can be reprogrammed into iPSCs, these cells have been shown to closely resemble embryonic stem cells (ESCs) in morphology, pluripotency gene expression, and growth characteristics [1]. Patient-derived iPSCs can be expanded and directed to differentiate into specific cell types, thereby enabling modeling of organ development and disease progression *in vitro*. This capacity makes iPSCs highly valuable for disease modeling, mechanistic investigation, and drug screening.

Over the past decade, iPSC-based systems have been applied to a wide spectrum of diseases. In developmental biology, patient-derived iPSCs have illuminated mechanisms of congenital syndromes and enabled testing of potential interventions. In neurology, they enable the modeling of Parkinson’s disease, Alzheimer’s disease, and other neurodegenerative disorders, capturing early cellular events inaccessible in postmortem tissues. In cardiovascular research, iPSC-derived cardiomyocytes have provided key insights into heart failure, arrhythmias, and cardiomyopathies [2]. These diverse applications highlight the versatility of iPSCs in bridging genetics and cell biology.

In cancer research, iPSC-based models have mainly followed two strategies. One strategy reprograms somatic cells from patients carrying hereditary cancer mutations into iPSCs, which are then differentiated into the relevant tissue types—an approach well suited to familial cancers and adaptable to starting materials such as peripheral blood mononuclear cells, bone marrow mononuclear cells, or fibroblasts. The other strategy reprograms tumor cells from patients directly into iPSCs; these cancer-derived iPSCs can retain tumorigenic traits and be used to trace tumor-initiating potential during differentiation [3]. These complementary approaches have expanded the cancer modeling toolkit, but their application to breast cancer has lagged behind.

**Figure 1.**
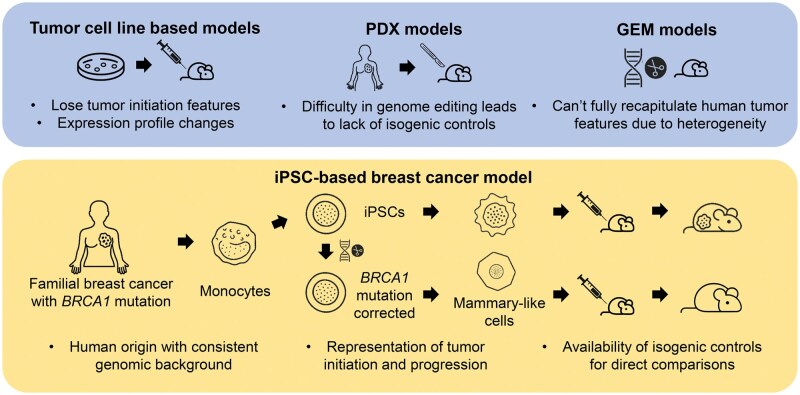
**Traditional breast cancer models versus the iPSC-based approach**. Comparison of conventional models—cell lines, PDX, and GEM—and their limitations, contrasted with an iPSC-based system that preserves human genetic background, models tumor initiation, and enables isogenic controls.

Breast cancer research has long relied on immortalized cell lines, patient-derived xenograft (PDX) models, and genetically engineered mouse (GEM) models. Each of these systems has provided invaluable insights, yet they also present limitations. Cell lines, while experimentally convenient, often lose tumor-initiating features and undergo gene expression drift during passaging. PDX models retain patient-specific features but typically lack isogenic controls, making it difficult to isolate the causal contribution of individual mutations. GEM models can reproduce certain genetic drivers, but the phenotypic outcomes of these mutations may differ from their counterparts in humans, limiting their ability to fully capture human tumor initiation and progression. iPSC-based models provide an opportunity to address these limitations by modeling tumorigenesis directly in a human developmental context while preserving both the genetic background and the temporal sequence of events (Fig. 1).

Our recent study [4] took up this challenge by establishing the first iPSC-based breast cancer model that recapitulates *BRCA1*-driven tumorigenesis. The *BRCA1* gene encodes a critical tumor suppressor protein that plays a fundamental role in DNA damage repair, and its germline mutations significantly increase the lifetime risk for breast cancer [5]. In our work, peripheral blood monocytes from a *BRCA1*-mutation carrier were reprogrammed into iPSCs and subjected to a stepwise mammary differentiation protocol. This approach generated mammary-like epithelial cells that exhibited both basal and luminal features, consistent with bipotent progenitors. When transplanted into cleared mammary fat pads, *BRCA1*-mutant cells progressed from premalignant states to invasive tumors. These tumors displayed histological hallmarks of triple-negative breast cancers. Importantly, isogenic controls were created through CRISPR/Cas9 correction of the *BRCA1* mutation, enabling direct comparisons between mutant and corrected lines in the same genetic background. Together, this system provides a controlled platform to study how BRCA1 deficiency drives breast cancer initiation and progression in human cells. Building on this foundation, integrative analyses revealed that BRCA1 represses the expression of S100P, a calcium-binding protein. Functional studies suggested that S100P promotes cancer stem cell programs through the RAGE/NF-κB signaling axis, whereas inhibition of this pathway reduced stemness and impaired tumor growth.

The significance of this work lies in several aspects. First, it demonstrates that iPSCs can be used to reconstruct the early steps of breast tumorigenesis in a way that complements existing models. Second, it introduces a system where isogenic comparisons are possible, allowing more confident causal inference regarding BRCA1 deficiency. Third, it identifies a potential regulatory pathway—S100P–RAGE—that connects BRCA1 loss to cancer stem cell plasticity, raising questions about whether modulation of this axis could affect therapeutic outcomes in *BRCA1*-associated cancers.

Overall, the iPSC-based platform could offer unique potential for studying early events underlying therapeutic response and resistance. By recapitulating early tumor-initiating stages from defined genetic backgrounds, these models enable controlled evaluation of how genetic context shapes drug sensitivity, stress adaptation, and the emergence of resistant phenotypes during lineage progression—extending beyond descriptive tumor biology to serve as predictive platforms for therapeutic testing and early-intervention strategies.

Looking forward, iPSC-derived breast cancer models can be further strengthened methodologically by (i) involving more donors and increasing donor diversity by incorporating iPSCs from additional patients, (ii) incorporating three-dimensional (3D) differentiation to better recapitulate ductal architecture and extracellular matrix interactions, (iii) integrating single-cell and spatial readouts to map lineage trajectories and clonal dynamics over time, and (iv) adding microenvironmental components (e.g., immune, fibroblast, and endothelial cells) to provide more physiologically relevant contexts for analyzing how *BRCA1*-associated tumor cells interact with their microenvironment and influence immune surveillance.

Beyond methodological refinement, future studies should also broaden the mechanistic scope of iPSC-based breast cancer models. Recent work has shown that enhancer DNA methylation linked to long-range chromatin interactions can serve as sensitive diagnostic biomarkers, underscoring the importance of 3D genome organization in cancer biology [6]. Our own work on somatic cell reprogramming has also shown that 3D genome organization can be regulated by the phase separation of key transcription factors such as OCT4, thereby influencing cell fate transitions and enhancing pluripotency gene expression [7]. These findings suggest that both chromatin architecture and phase separation are powerful regulators of gene expression programs and may play important roles in cell fate transitions and disease. In the context of *BRCA1*-associated breast cancer, it will be important to investigate whether cancer stem cell-related pathways are similarly shaped by changes in higher-order chromatin structure and condensate dynamics. Such mechanistic studies could illuminate how BRCA1 loss couples with epigenetic and biophysical processes to drive tumor initiation and therapeutic resistance.

Turning to generalizability and applicability, the overall workflow—patient-derived iPSCs, isogenic editing, directed differentiation—readily generalizes to other predisposition settings such as *BRCA2*- and *TP53*-associated cancers, as well as malignancies driven by mismatch-repair deficiency. However, cancer-type-specific lineage programs, cell-of-origin differences, stromal requirements, reprogramming efficiency, and residual epigenetic memory will necessitate tailored differentiation protocols and microenvironmental components.
